# Reducing sources of variance in experimental procedures in in vitro research

**DOI:** 10.12688/f1000research.73497.1

**Published:** 2021-10-12

**Authors:** Igor Fischer, Maria Victoria Martinez-Dominguez, Daniel Hänggi, Ulf Kahlert

**Affiliations:** 1 Department of Neurosurgery, Heinrich Heine University Düsseldorf, Düsseldorf, 40225, Germany; 2 Clinic for General, Visceral, Vascular, and Transplant Surgery, Otto-von-Guericke-Universität Magdeburg, Magdeburg, 39106, Germany

**Keywords:** reproducibility, standardization, quality control, in vitro research

## Abstract

**Background:** Lack of reproducibility in preclinical research is a problem posing ethical and economic challenges for biomedical science. Various institutional activities from society stakeholders of leading industry nations are currently underway to improve the situation. Such initiatives usually attempt to tackle high-level organisational issues and do not typically focus on improving experimental approaches per se. Addressing these is necessary in order to increase consistency and success rates of lab-to-lab repetitions.

**Methods**: In this project, we statistically evaluated repetitive data of a very basic and widely applied lab procedure, namely quantifying the number of viable cells. The purpose of this was to appreciate the impact of different parameters and instrumentations that may constitute sources of variance in this procedure.

**Conclusion: **By comparing the variations of data acquired under two different procedures, featuring improved stringency of protocol adherence, our project attempts to propose guidelines on how to reduce such variations. We believe our work can contribute to tackling the repeatability crisis in biomedical research.

## 1. Introduction

*In vitro* work is the fundament of every wet lab project, both in academic research as well as in industry product development. With the recently emerging global move to reduce, refine, or replace animal research, ^1^ the impact of cell model research on total research output is expected to increase even further.

Some areas of biomedical research suffer from insufficient success rates to replicate or repeat core findings of previous observations. A survey amongst scientists recently highlighted that the community is not only well aware of this problem, but also confirmed that personal experiences in this issue are common. ^11, 2, 12^ A standard *in vitro* method for drug development or genetic studies is the quantification of cellular growth over time, under different micro-environmental conditions. For this basic assay, on which to some extent almost all global oncology blockbusters of today’s market are based on, inconsistencies in repeating research results have been noticed. ^8, 6, 13^ In response this this, leading science organizations, in cooperation with respective policy makers, scientific publishing houses, and other society stakeholders, have initiated institutionally funded campaigns to tackle these issues, such as the cancer reproducibility project, to mention only one. ^4, 15^ Moreover, improved and increased standardization in wet lab research is proposed to be a powerful strategy to increase academic science repeatability. ^3, 9, 5^

Accurate cell counting is crucial in laboratories for research purposes, medical diagnosis and treatment. As an example, a precise knowledge of the number of cells in blood samples of a patient could be crucial in the determination of the cause of disease. If the analytical performance is uncertain or deviates from other methodologies, the method should not be implemented.

Currently, the main methods for cell counting are manual counting or automated cell counting. Each of these specific methods comes with its advantages and disadvantages. On one hand, manual counting relies on the experimenter, microscopes and counting chambers, seemingly causing a higher error in repeatability, which only increases with the number of samples to analyze. Additionally, it is laborious and time-consuming. ^16, 7^ Increasingly, manual counting is being replaced by automated cell counting, as its in the case of blood cell counting, where it present several additional difficulties, such as the distinguishing in size or morphology of blood cell types, which is crucial for a correct identification. ^14^

Automated counting is fast, efficient and does not include a human error beyond the process of sample preparation. Nevertheless, accuracy and sensitivity are lost after a long-time use of the machine and more and more often calibration steps are needed. ^7^ In addition, cell automatic counting is not well suited for volumes lower than $10^{5}$ and larger than $10^{7}$ cells, under or overestimating the cell number and therefore the clinical reliability.

In this study we investigate the repeatability and variance of results in experiments involving human operator and automatic instrumentation-based quantification of cells. In order to quantify the variance and identify its sources, we set up an experiment in preparing the stem cell suspension and counting the cells in it, both viable and dead. Our experiment included three researchers with different amount of experience, who prepared and counted viable and dead tumor stem cells. Moreover, by comparing the variances in data acquired under two different procedures, our project attempts to propose guidelines how to reduce them. We believe our work supports ambitions to tackle biomedical repeatability crisis by promoting the power of standard operating procedures to reduce variance in early stage wet lab experimentation.

## 2. Material and methods

### 2.1 Cell model and cell preparation

As our cell model, we selected a state of the art 3D cell culture (the familiar brain tumor stem cell line NCH644 generously provided by Prof. Herold-Mende, Heidelberg, Germany). The cells were cultured as suspension under neurosphere conditions as described elsewhere. ^10^ After incubation, each experimenter prepared a cell suspension for counting, by the standard wetlab procedure (Supplementary file SOP-cellPreparation.pdf). A total volume of 1 ml of cells from each suspension was taken for counting by every researcher: the one who prepared the cells and the two other scientists. Compared to the first run, essential specifications that were added to the guidelines to perform the experimental procedure are: timing of each step, amount of trypsin times of pipetting for cell separation and, most importantly, familiarization with the procedure. The cells, viable and dead, were counted in two different ways: manually, under the microscope, and automatically using a specialized cell counting machine.

### 2.2 Cell quantification

**Human operator counting:** Three researchers with different levels of wetlab experience were involved in the experiments: A senior, experienced researcher (UKD), somewhat out of daily practice; a MS student of molecular biology (VM) who works in the wetlab on an almost daily basis; and a computer scientist and a complete novice to wetlab research (IF). For manual counting, the haemocytometer, also known commonly as Neubauer-improved chamber with 0.1 μ l capacity was used. The viable (defined as white) and dead (blue) cells were counted under the microscope in four quadrants using brightfield microscopy with a 10× objective. Each researcher counted cells in two different samples (two fields of the same Neubauer chamber) from the same suspension. Each sample was counted by all three researchers, resulting in 48 counts per suspension: 3 researchers × 2 samples × 4 quadrants × 2 types of cells, viable and dead. As three different suspensions were prepared, this led to 144 different counts per experiment.

The cell concentration (cells/ml) was computed by multiplying the number of cells, summed over the quadrants, by 10,000 ( =1000μ l/ml/0.1 μ l) and dividing by the number of quadrants (4).

**Automated counting** was done using Guava ^®^ Muse ^®^ Cell Analyzer, Luminex second generation, SN 7200121445, Luminex, Germany, using Muse ^®^ Count & Viability Kit (MCH600103) according to manufacturer descriptions. Both the viability and the cell size (fragmented dead cells versus alive cells) parameters were left as default. Each researcher's cell suspension was measured three times in a row both with the Neubauer chamber and the Cell Analyzer.

The above experiment was performed twice: first, without explicitly establishing the standard operating procedures (SOPs) and, weeks later, by adhering to SOPs written for that purpose as part of the labs quality control system. The SOP can be found in the supplementary data of this article (Supplementary data File SOP-cellCounting.pdf).

### 2.3 Statistics

The homogeneity of cell counts in the Neubauer chamber was checked using the $\chi^{2}$ -test. Linear regression was used to model viability of cells over time. Uniformity of cell counts between the three researchers was tested using ANOVA and post-hoc t -test. F -test was used for comparing variances in the data. All computations were performed in Python, using numpy and scipy.stats modules.

## 3. Results

**Figure 1.**
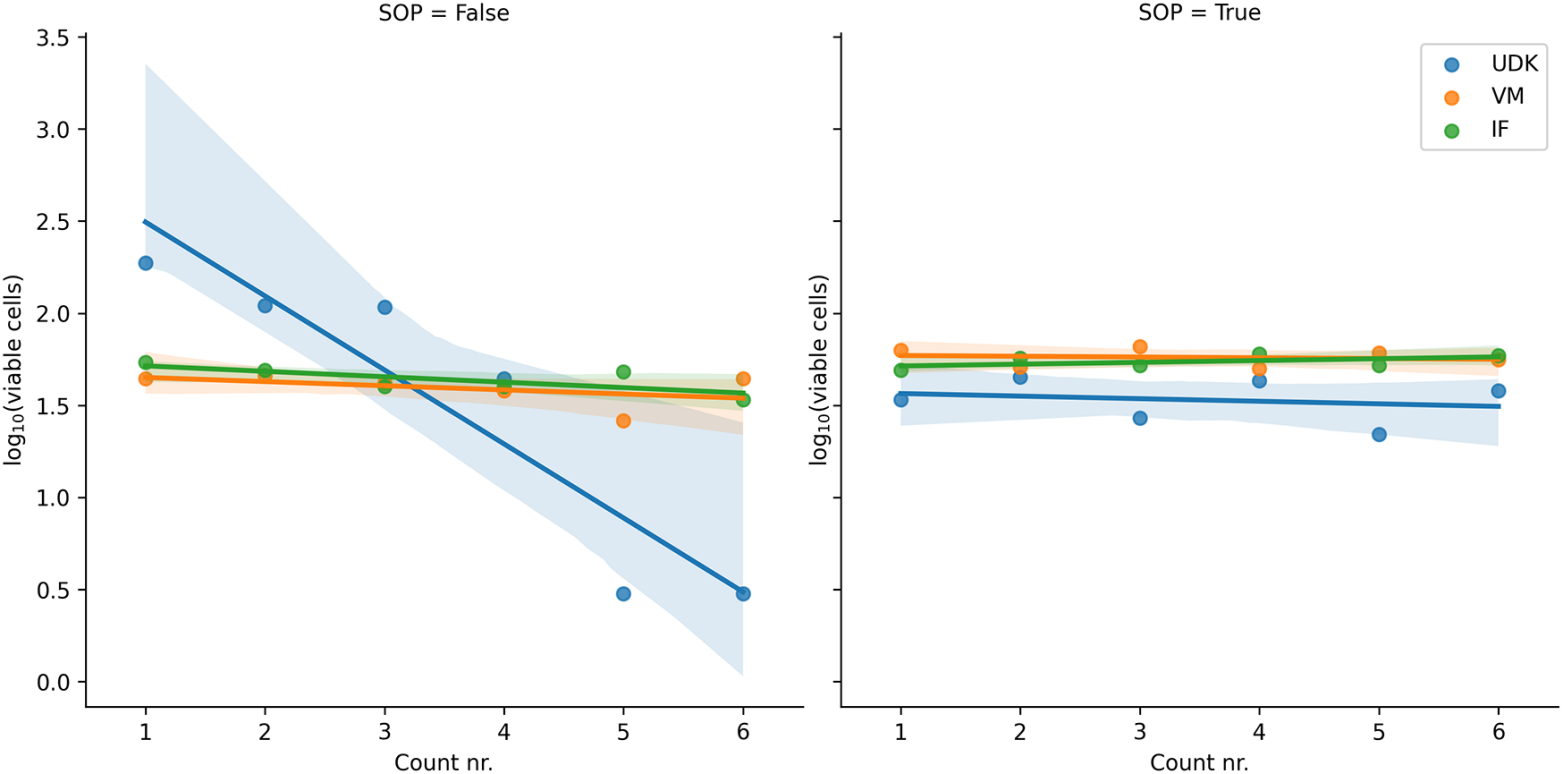
Without adhering to the SOPs (left), the cells used in one solution (UDK) were highly volatile and kept dying during counting. In the repeated experiment, done according to the SOPs, all solutions remained stable with time. The ordinal count number (1st counting, 2nd counting etc.) is shown on the *x*-axis and the logarithm of the number of cells counted on the *y*-axis.

In the first experiment, without the SOPs, the NCH-644 cells from which the first suspension was made were not viable enough and kept dying rapidly during counting. Consequently, the cell counts among the researchers differed significantly, from around 500,000/ml at the beginning of the counting down to 140,000 at the end ( $p{<10}^{-20}$ , Figure 1). For further analysis, we excluded this first suspension and used a different cell culture to prepare new solutions.

To quantify the homogeneity of the suspensions, we performed $\chi^{2}$ -test of homogeneity of manual counts in the four quadrants, separately for each counting researcher. Altogether, inhomogeneous cell numbers ( p<0.05 ) were found in 25% of the cases (9 out of 36). Comparing cell counts between different researchers, separately for each quadrant, showed no significant differences, except in one case when one researcher (IF) counted significantly more dead cells ( $p{<10}^{-4}$ ).

Using manual counting, the total cell concentration, viable + dead, varied between 105,000 and 192,500 cells/ml (mean = 137,000, sd = 28,000). Automated counting produced significantly higher numbers ( p=0.0013 ): between 163,700 and 317,500 cells/ml, with mean = 271,100 and sd = 55,900. Despite the difference in counts, the coefficient of variation (cv = sd/mean) was almost identical for manual and automated counting, 0.205 vs. 0.206, respectively. In both cases, the mean counts were similar, regardless of the researcher who prepared the solution ( p=0.57 for manual counting and p=0.41 for automated counting). But, the solution prepared by VM was significantly more homogenous, having lower variance than the one prepared by IF ( F -test, p=0.013 ).

A similar pattern, with an even greater discrepancy between manual and automated counting could be observed when taking only viable cells into account. For manual counting, the variance of the viable cells is not as large as for the total number of cells. Interestingly, automated counting found a very low variance of dead cells. With manual counting, the number of viable cells varied between 65,000 and 135,000 cells/ml, with mean = 105,200, sd = 18400, and cv = 0.168.

In the second run of the experiment, performed according to the SOPs, all cells were viable and could be considered for counting. it was observed that the homogeneity of the cell counts in different quadrants of the Neubauer chamber did not improve: We again encountered 9 out of 36 cases where the numbers of cells in the quadrants differed significantly ( *p* < 0.05), according to the chi-squared test.

**Figure 2.**
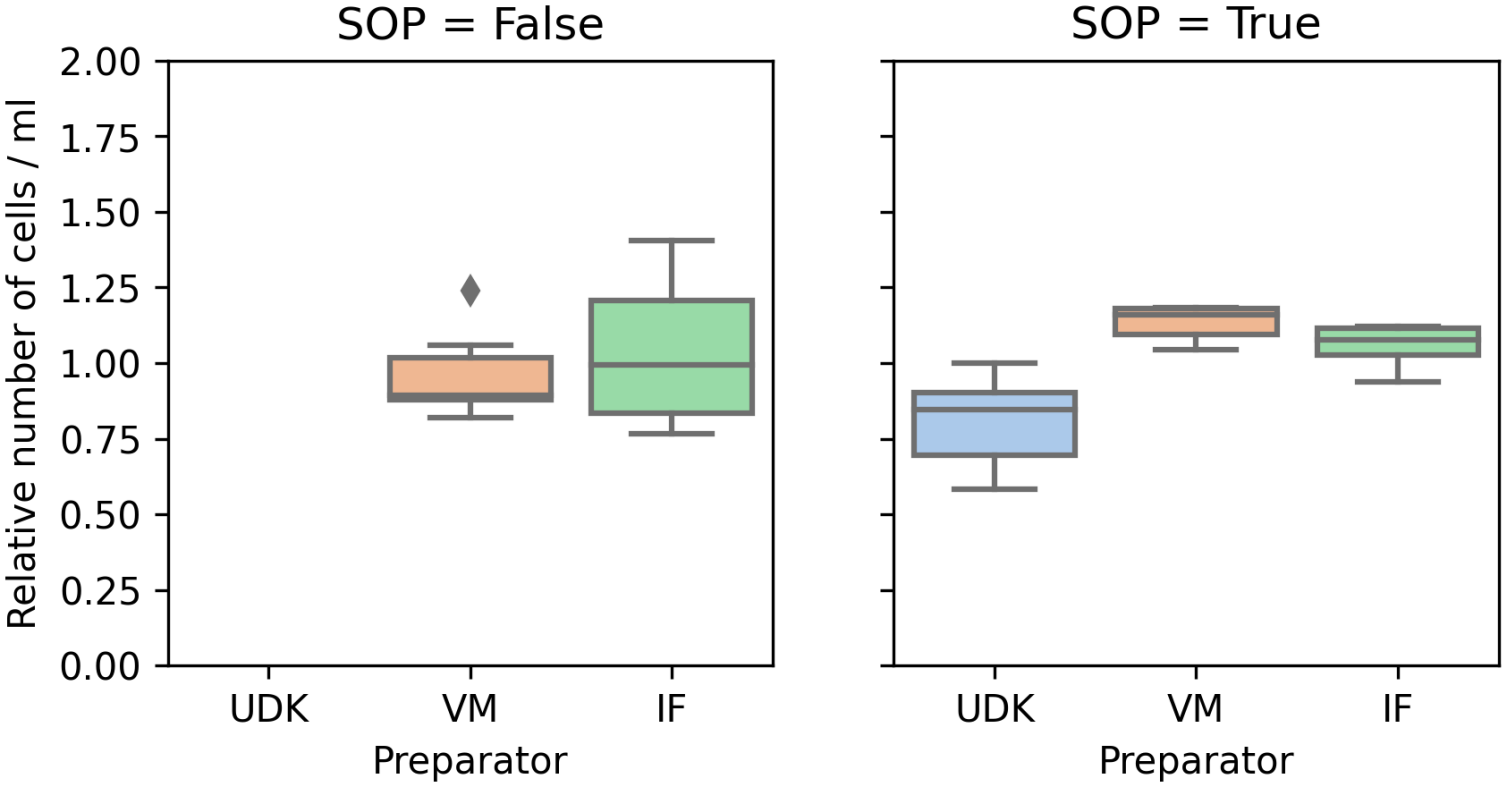
Manual counting: Without adhering to the SOPs (left), the coefficient of variance in the total number of cells was significantly higher then when the experiment was performed in accordance to the SOPs (right). To ensure comparability between experiments, the *y*-axis shows the cell count scaled by the mean of the cell count in the respective experiment.

**Figure 3.**
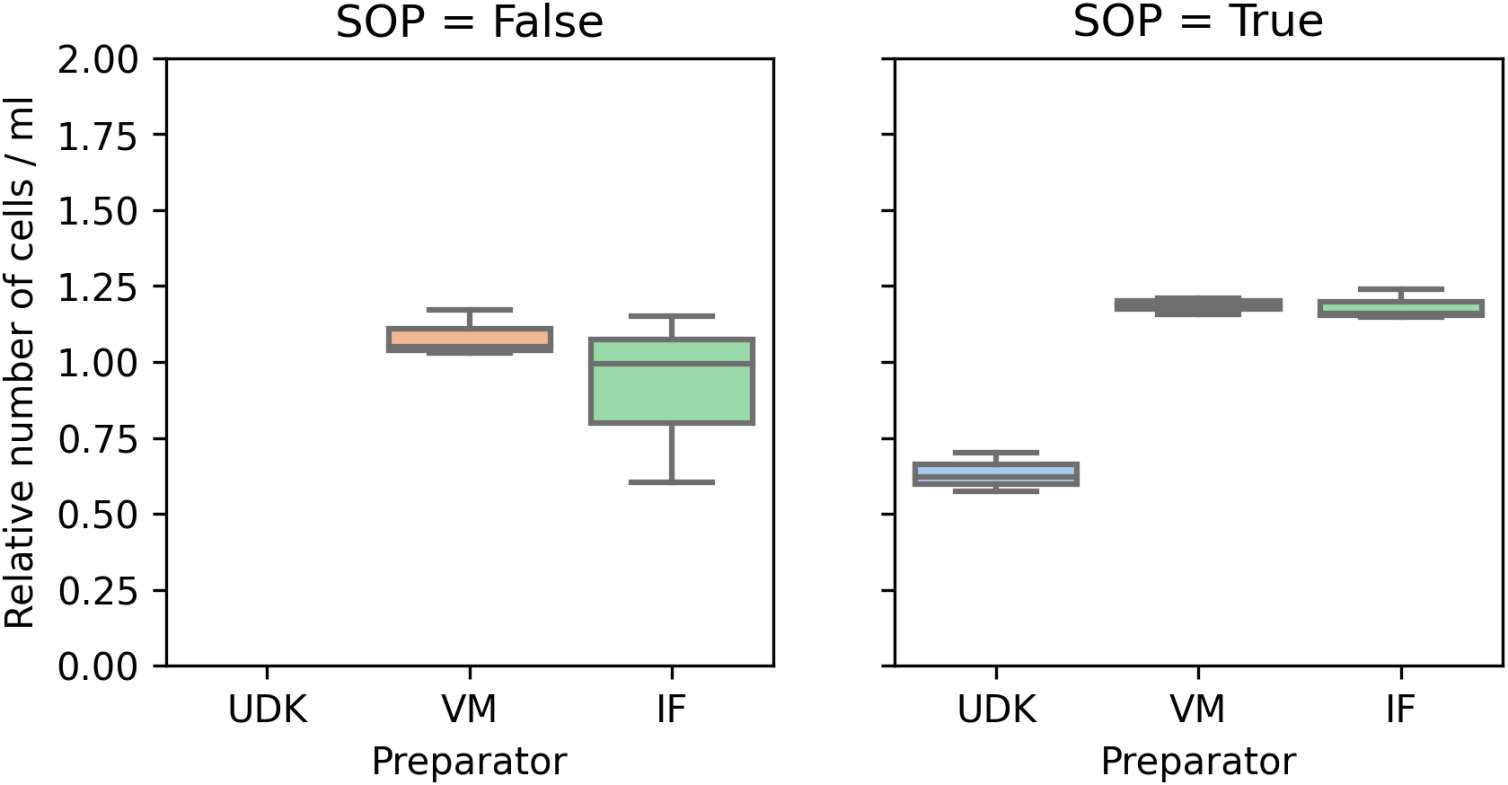
Automatic counting: Here, too, the coefficient of variance in the total number of cells was significantly higher when the experiment was performed without adhering to the SOPs (left). Again, as in Figure 2, the cell counts are scaled to ensure comparability.

Comparing the counts in the two runs, for the two researchers whose cells were viable in the first run, performing the experiment by the SOPs significantly reduced the variance in the counts, both manual and automated. In the first run, the coefficient of variation (cv) for the manual count was in the range 0.16 to 0.24, and 0.07 to 0.30 for the automated counting. In the second run, the cv for manual counting fell to 0.05 to 0.07, and to 0.02 to 0.04 for the automated counting ( Figures 2 and 3).

**Figure 4.**
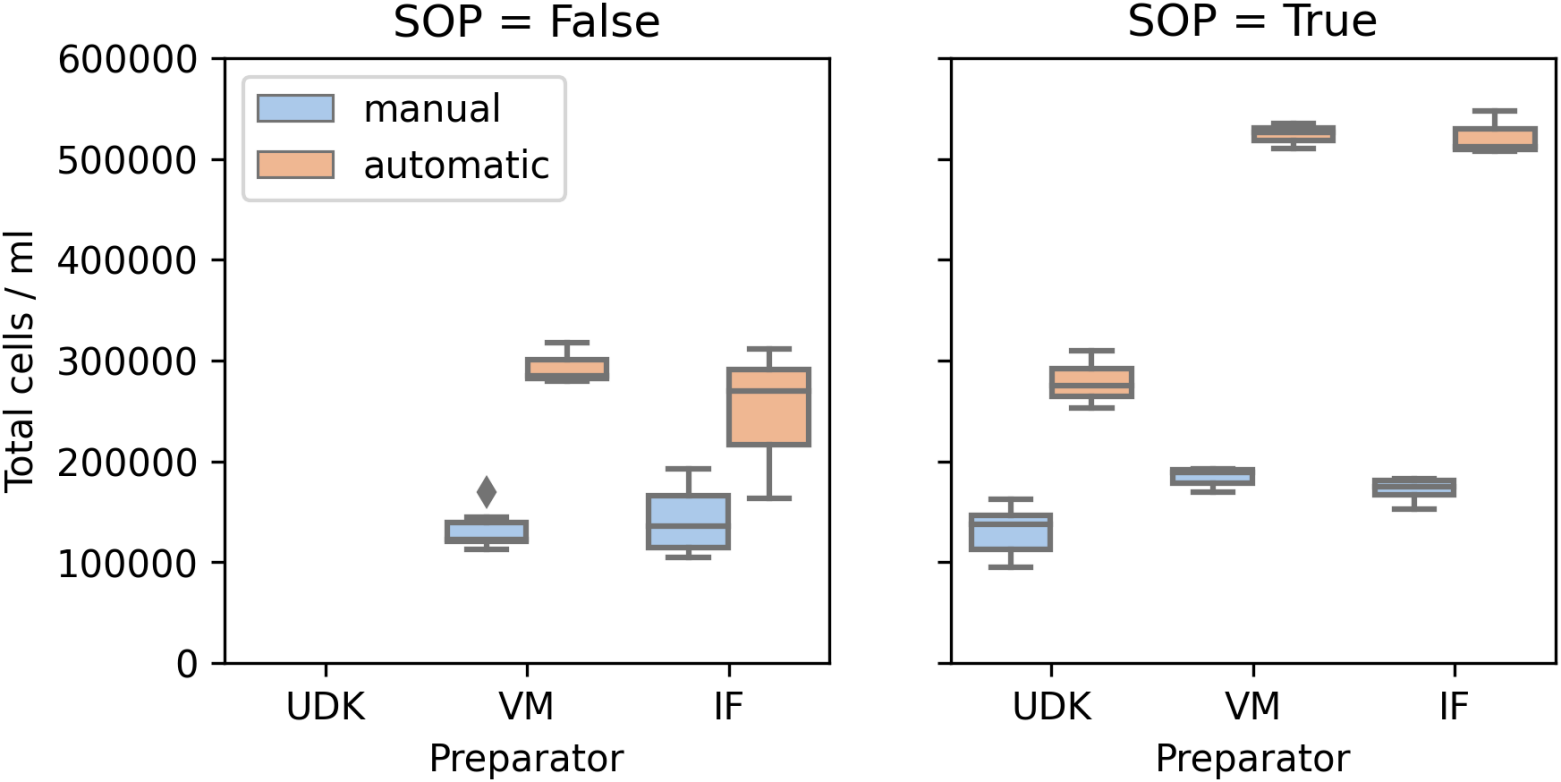
Comparison of the absolute numbers of cells in the solution. Cell Analyzer consistently counts more cells than the researchers using the microscope.

Surprisingly, manual and automated counting led to significantly different numbers, in both runs ( Figure 4). While for the manual counting the total cell numbers were consistently in the range 100,000 – 200,000, the counting machine found 150,000 – 300,000 in the first run and 250,000 – 550,000 in the second. Another point worth noticing is that the number of live and dead cells varied between the machine operators. As the machine requires manual adjustment of threshold for detecting dead cells, we believe this to be one source of variation.

## 4. Discussion

Given the very wide dissemination of the applied experimental procedure, this project presents—to the best of our knowledge—the hitherto first dedicated calculation of sources of variance on cell quantification between automated and manual cell counting in suspension stem cell lines. Although the setup per se is rather simple nature, we are convinced the drawn conclusions are of significance for the general readership.

Research laboratories need to do cell counting on a daily basis. Parameters as precision, accuracy, time-consumption and reliability are critical for a good performance. With this perspective, this study compared two forms of a widely used basic lab procedure: cell quantification.

To evaluate the performance of automated and manual counts, statistical analyses have been made to address the accuracy and repeatability to benchmark one against other method. Careful preparation of the cell culture proved critical. In our case, the experienced researcher (UDK) noticed already during counting that the numbers of live cells were decaying, but this experiment was also specifically set up to identify such problems. The suspicion was confirmed in further counting by the other two researchers (VM, IF). However, due to time and personnel constraints, multiple counting of a same probe is uncommon. It is therefore conceivable that a less experienced or less attentive (unsuspecting) researcher would fail to notice this problem. Due to exponential cell growth, variations in the starting number of cells grow much larger after incubation. For our manual counting of viable cells, the initial variation of 16% would increase to 44% after three mitoses. The used cell model is in general range for cell proliferation index of many cancer cells (18–36 hours) and therefore our results may be representable for scenarios when working with other cell models. When the manual countings between different researchers was compared between quadrants, one researcher (IF) counted significantly more dead cells ( $p{<10}^{-4}$ ). The other two researchers’ guess is that he, due to lack of experience, counted cell debris as dead cells. Adhering to the SOPs led to more consistent results. The second time, the procedure was better prepared and performed more quickly, this indicates that the duration of the experiment plays a role.

Independently of the human operator that prepared the initial cell suspension, we noticed severe difference in absolute quantification of cells (viable and dead) between manual and automatic counting. From our data, this somewhat surprising outcome cannot be explained scientifically. Although our instrument handling and counting setup was performed according manufacturer instructions, it remains possible, albeit somewhat speculative, that the gating setting caused the Cell Analyzer to misclassify debris or cell doublets as single cells. Moreover, comparing quantification outcomes based on biological assays that exploit same property of the cells (cell membrane integrity), but use different reporter signal (human counting is based on colorimetric, whereas an automatic counting is based on fluorescence), is likely another cause of the differences. Since in vitro cell growth assays usually compare different genetic conditions or environmental stressors, absolute quantification is less important than relative quantification between different test conditions. As long as only relative cell growth/cell dying is of concern, experiments can be set up manually and checked for result stability. Automated counting is not an impediment as long as the same counting method is used when comparing data. This is especially important in lab-to-lab validation, to ensure comparable results.

However, absolute cell number is important in order to define optimal cell plating density at the beginning of the experiment: Sufficient, but not too dense cell-cell contact/confluence grade is a critical factor which impacts the cells’ growth rate, so cells need to be plated in a density that allows exponential growth under control conditions over the entire period of the experiment. Therefore, we suggest to acquire an individual standard growth rate for each cell model to be studied. Our data show that it is highly relevant that the exact same procedure—counting for plating and indirect method for readout at the end of experiment such as MTT/cell TiterGlo—is applied for this “preparatory analysis” as for the actual experiment. Lastly, and of well-known nature, our experiment ignores benefits of automatic counting in the context of larger sample series, such as less burden on human operator concentration as well as acceleration of the experiment.

Importantly, adhering the execution to the SOPs, which define maximum time of the execution, familiarization with the SOP, addition of trypsin for a specific time in the incubator and specification of number of times of pipetting for cell separation, led to more stable, in our viewpoint consistent and repeatable results. Although our experiments were performed on a small scale, adhering to the SOPs showed a significant improvement in the stability of the results. We therefore suggest devising such SOPs and enforcing the adherence in wetlab research.
