# Reducing sources of variance in experimental procedures in in vitro research

**DOI:** 10.12688/f1000research.73497.2

**Published:** 2022-01-07

**Authors:** Igor Fischer, Maria Victoria Martinez-Dominguez, Daniel Hänggi, Ulf Kahlert

**Affiliations:** 1 Department of Neurosurgery, Heinrich Heine University Düsseldorf, Düsseldorf, 40225, Germany; 2 Clinic for General, Visceral, Vascular, and Transplant Surgery, Otto-von-Guericke-Universität Magdeburg, Magdeburg, 39106, Germany

**Keywords:** reproducibility, standardization, quality control, in vitro research

## Abstract

**Background:** Lack of reproducibility in preclinical research poses ethical and economic challenges for biomedical science. Various institutional activities by society stakeholders of leading industrialised nations are currently underway with the aim of improving the situation. Such initiatives are usually concerned with high-level organisational issues and typically do not focus on improving experimental approaches per se. Addressing these is necessary in order to increase consistency and success rates of lab-to-lab repetitions.

**Methods**: In this project, we statistically evaluated repetitive data of a very basic and widely applied lab procedure, namely quantifying the number of viable cells. The purpose of this was to assess the impact of different parameters and instrumentations which may constitute sources of variance in this procedure.

**Conclusion: **By comparing the variability of data acquired under two different procedures, featuring improved stringency of protocol adherence, our project attempts to identify the sources and propose guidelines on how to reduce such fluctuations. We believe our work can contribute to tackling the repeatability crisis in biomedical research.

## 1. Introduction

*In vitro* work is the fundament of every wet lab project, in academic research as well as in industry product development. With the recently emerging global move to reduce, refine, or replace animal research, ^1^ the impact of cell model research on total research output is expected to increase even further.

Some areas of biomedical research suffer from insufficient success rates to replicate or repeat core findings of previous observations. A survey amongst scientists recently highlighted that the community is not only well aware of this problem, but also confirmed that personal experiences with this issue are common. ^11, 2, 12^ A standard *in vitro* method for drug development or genetic studies is the quantification of cellular growth over time, under different micro-environmental conditions. For this basic assay, on which to some extent almost all of today’s successful substances used in oncology are based, inconsistencies have been noticed when trying to repeat research results. ^8, 6, 13^ In response this this, leading science organizations, in cooperation with respective policy makers, scientific publishing houses, and other society stakeholders, have initiated institutionally funded campaigns to tackle these issues, such as the cancer reproducibility project, to mention only one. ^4, 15^ Also, improved and increased standardization in wet lab research is suggested as a powerful strategy to increase repeatability. ^3, 9, 5^

Accurate cell counting is crucial in laboratories for research purposes, medical diagnosis and treatment. As an example, precise knowledge of the number of cells in blood samples of a patient could be crucial in the determination of the cause of disease. If the analytical performance is uncertain or deviates from other methodologies, the method should not be used.

Currently, the main methods for cell counting are manual counting and automated cell counting. Each of these specific methods comes with its advantages and disadvantages. On one hand, manual counting relies on the experimenter, microscopes and counting chambers, apparently causing a higher error in repeatability, which only increases with the number of samples to analyze. Additionally, it is laborious and time-consuming. ^16, 7^ Increasingly, manual counting is being replaced by automated cell counting, as in the case of blood cell counting, where it presents several additional difficulties, such as the distinguishing size or morphology of blood cell types, which is crucial for correct identification. ^14^

Automated counting is fast, efficient and does not include a human error beyond the process of sample preparation. Nevertheless, accuracy and sensitivity are lost after a long-time use of the machine and more and more often calibration steps are needed. ^7^ In addition, cell automatic counting is not well suited for volumes lower than $10^{5}$ and larger than $10^{7}$ cells, leading to either underestimating or overestimating the total cell numbers and therefore limiting the clinical reliability.

In this study we investigate the repeatability and variance of results in experiments involving human operator and automatic instrumentation-based quantification of cells. In order to quantify the variance and identify its sources, we set up an experiment in preparing the stem cell suspension and counting the cells in it, both viable and dead. Our experiment included three researchers with different amount of experience, who prepared and counted viable and dead tumor stem cells. In addition to comparing the variances in data acquired under two different procedures, our project attempts to propose guidelines how to reduce them. We believe our work supports ambitions to tackle biomedical repeatability crisis by promoting the power of standard operating procedures to reduce variance in early stage wet lab experimentation.

## 2. Material and methods

### 2.1 Cell model and cell preparation

As our cell model, we selected a state of the art 3D cell culture (the familiar brain tumor stem cell line NCH644 generously provided by Prof. Herold-Mende, Heidelberg, Germany). The cells were cultured as suspension under neurosphere conditions as described elsewhere. ^10^ After incubation, each experimenter prepared a cell suspension for counting, by the standard wetlab procedure (Supplementary file SOP-cellPreparation.pdf). A total volume of 1 ml of cells from each suspension was taken for counting by every researcher: the one who prepared the cells and the two other scientists. The cells, viable and dead, were counted in two different ways: manually, under the microscope, and automatically using a specialized cell counting machine.

### 2.2 Cell quantification

**Human operator counting:** Three researchers with different levels of wetlab experience were involved in the experiments: A senior, experienced researcher (UDK), somewhat out of daily practice; a MS student of molecular biology (VM) who works in the wetlab on an almost daily basis; and a computer scientist and a complete novice to wetlab research (IF). For manual counting, the haemocytometer, also known commonly as Neubauer-improved chamber with 0.1 *μ*l capacity was used. The viable (defined as white) and dead (blue) cells were counted under the microscope in four quadrants using brightfield microscopy with a 10× objective. Each researcher counted cells in two different samples (two fields of the same Neubauer chamber) from the same suspension. Each sample was counted by all three researchers, resulting in 48 counts per suspension: 3 researchers × 2 samples × 4 quadrants × 2 types of cells, viable and dead. As three different suspensions were prepared, this led to 144 different counts per experiment.

The cell concentration (cells/ml) was computed by multiplying the number of cells, summed over the quadrants, by 10,000 (= 1000 *μ*l / ml / 0.1 *μ*l) and dividing by the number of quadrants (4).

**Automated counting** was done using Guava ^®^ Muse ^®^ Cell Analyzer, Luminex second generation, SN 7200121445, Luminex, Germany, using Muse ^®^ Count & Viability Kit (MCH600103) according to manufacturer descriptions. Both the viability and the cell size (fragmented dead cells versus alive cells) parameters were left as default. Each researcher's cell suspension was measured three times in a row both with the Neubauer chamber and the Cell Analyzer.

The above experiment was performed twice: first, without explicitly establishing the standard operating procedures (SOPs) and, weeks later, by adhering to SOPs written for that purpose as part of the labs quality control system. Compared to the first run, essential specifications that were added to the guidelines to perform the experimental procedure are: timing of each step, amount of trypsin times of pipetting for cell separation and, most importantly, familiarization with the procedure. The SOP can be found in the supplementary data of this article (Supplementary data File SOP-cellCounting.pdf).

### 2.3 Statistics

The homogeneity of cell counts in the Neubauer chamber was checked using the *χ* ^2^-test. Linear regression was used to model viability of cells over time. Uniformity of cell counts between the three researchers was tested using ANOVA and post-hoc *t*-test. *F*-test was used for comparing variances in the data. All computations were performed in Python, using numpy and scipy.stats modules.

## 3. Results

**Figure 1.**
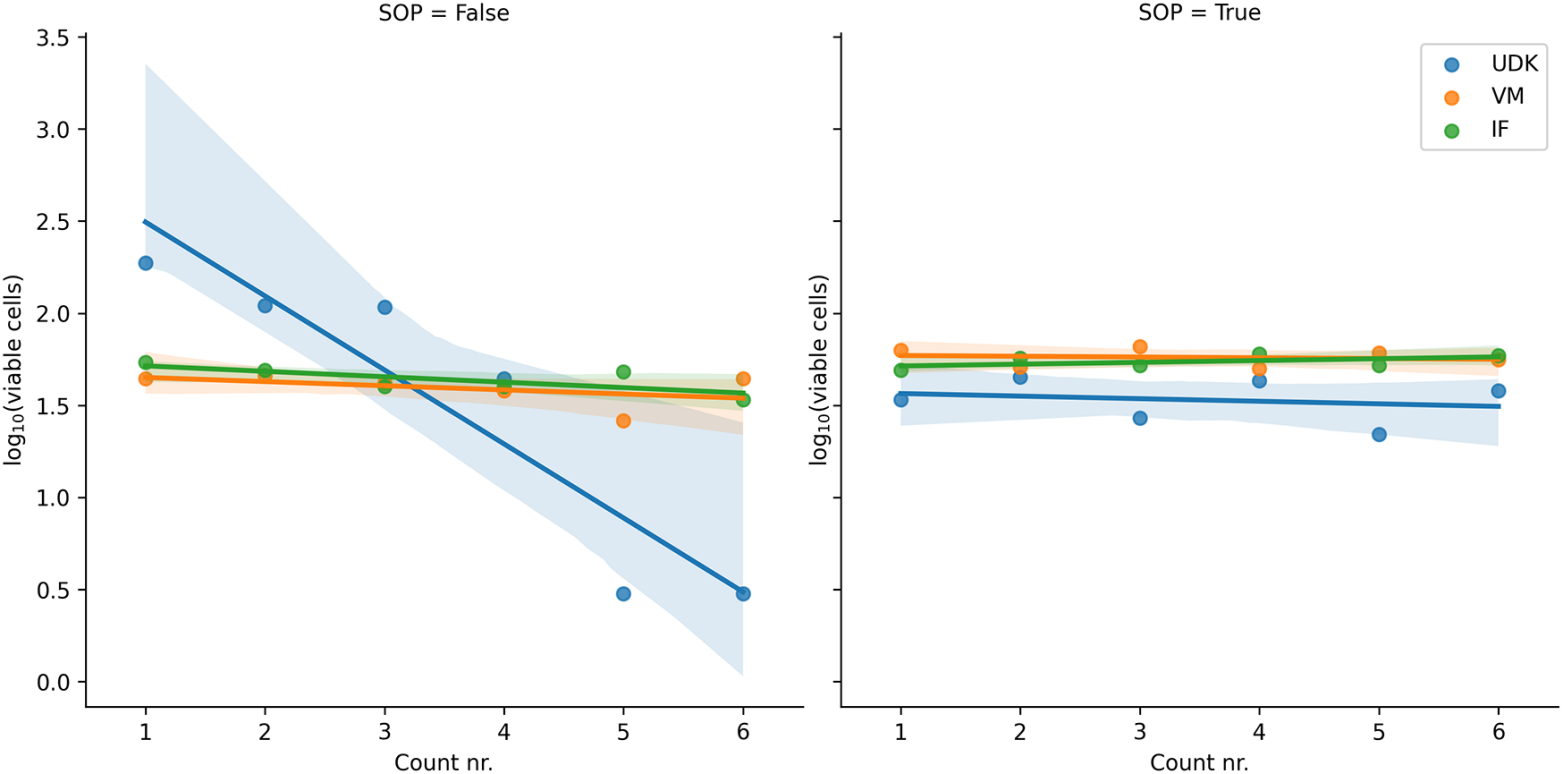
Without adhering to the SOPs (left), the cells used in the first prepared solution (UDK) were highly volatile and kept dying during counting. In the repeated experiment, done according to the SOPs, all solutions remained stable with time. The ordinal count number (1st counting, 2nd counting etc.) is shown on the *x*-axis and the logarithm of the number of cells counted on the *y*-axis.

In the first experiment, without the SOPs, the NCH-644 cells from which the first suspension was made (prepared by UDK) were not viable enough and kept dying rapidly during counting. Consequently, the cell counts among the researchers differed significantly, from around 500,000/ml at the beginning of the counting down to 140,000 at the end ( *p* < 10 ^−20^, Figure 1). Although the way the experiment was conceived did not allow for establishing the cause with certainty, we assume that a toxic agent - perhaps the alcohol used for cleaning the equipment - somehow contaminated the culture. For further analysis, we excluded this first suspension and used a different cell culture to prepare new solutions. The second solution was prepared by VM and the third by IF. Special care was taken to avoid contamination.

To quantify the homogeneity of the suspensions, we performed *χ* ^2^-test of homogeneity of manual counts in the four quadrants, separately for each counting researcher. Altogether, inhomogeneous cell numbers ( *p* < 0.05) were found in 25% of the cases (9 out of 36). Comparing cell counts between different researchers, separately for each quadrant, showed no significant differences, except in one case when one researcher (IF) counted significantly more dead cells ( *p* < 10 ^−4^).

Using manual counting, the total cell concentration, viable + dead, varied between 105,000 and 192,500 cells/ml (mean = 137,000, sd = 28,000). Automated counting produced significantly higher numbers ( *p* = 0.0013): between 163,700 and 317,500 cells/ml, with mean = 271,100 and sd = 55,900. Despite the difference in counts, the coefficient of variation (cv = sd/mean) was almost identical for manual and automated counting, 0.205 vs. 0.206, respectively. For both manual and automated counting, the differences in the mean counts were insignificant, regardless of the researcher who prepared the solution ( *p* = 0.57 for manual counting and *p* = 0.41 for automated counting). But, the solution prepared by VM was significantly more homogenous, having lower variance than the one prepared by IF ( *F*-test, *p* = 0.013).

A similar pattern could be observed when taking only viable cells into account. Here, too, automated counting resulted in noticeably higher numbers. With manual counting, the number of viable cells varied between 65,000 and 135,000 cells/ml, with mean = 105,200, sd = 18400, and cv = 0.175. Automated counting found between 125,200 and 271,700 cells, with mean = 212,500, sd = 49,300, and cv = 0.232. Interestingly, automated counting found a much lower variance of dead cells (38,400 – 80,000, mean = 58,700, sd = 20,900, cv = 0.35), than manual counting (2,500 – 80,000, mean = 31,900, sd = 25,500, cv = 0.8).

In the second run of the experiment, performed according to the SOPs, all cells were viable and could be considered for counting. It was observed that the homogeneity of the cell counts in different quadrants of the Neubauer chamber did not improve: We again encountered 9 out of 36 cases where the numbers of cells in the quadrants differed significantly ( *p* < 0.05), according to the *χ* ^2^-squared test.

**Figure 2.**
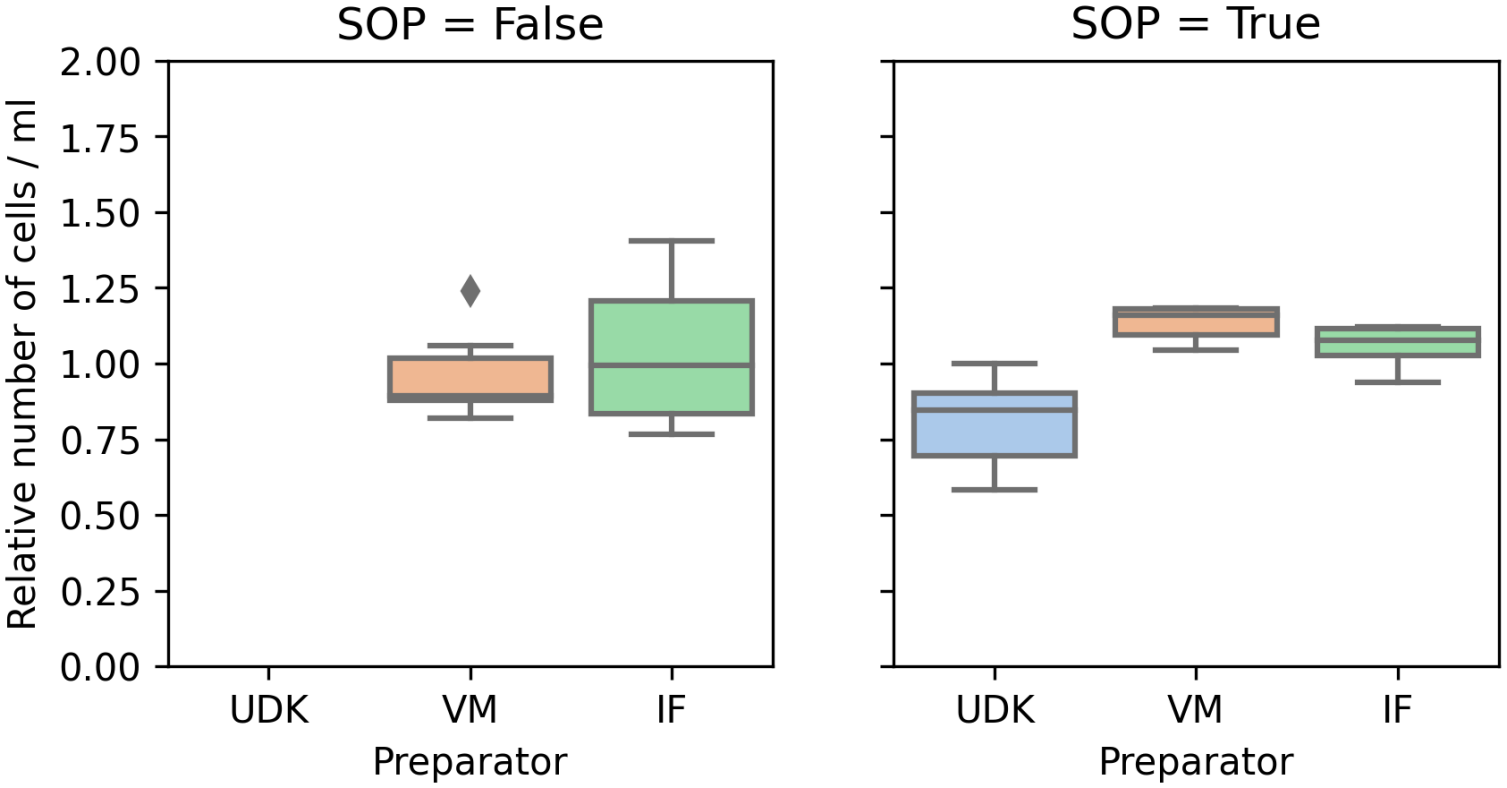
Manual counting: Without adhering to the SOPs (left), the coefficient of variance in the total number of cells was significantly higher than when the experiment was performed in accordance to the SOPs (right). To ensure comparability between experiments, the *y*-axis shows the cell count scaled by the mean of the cell count in the respective experiment.

**Figure 3.**
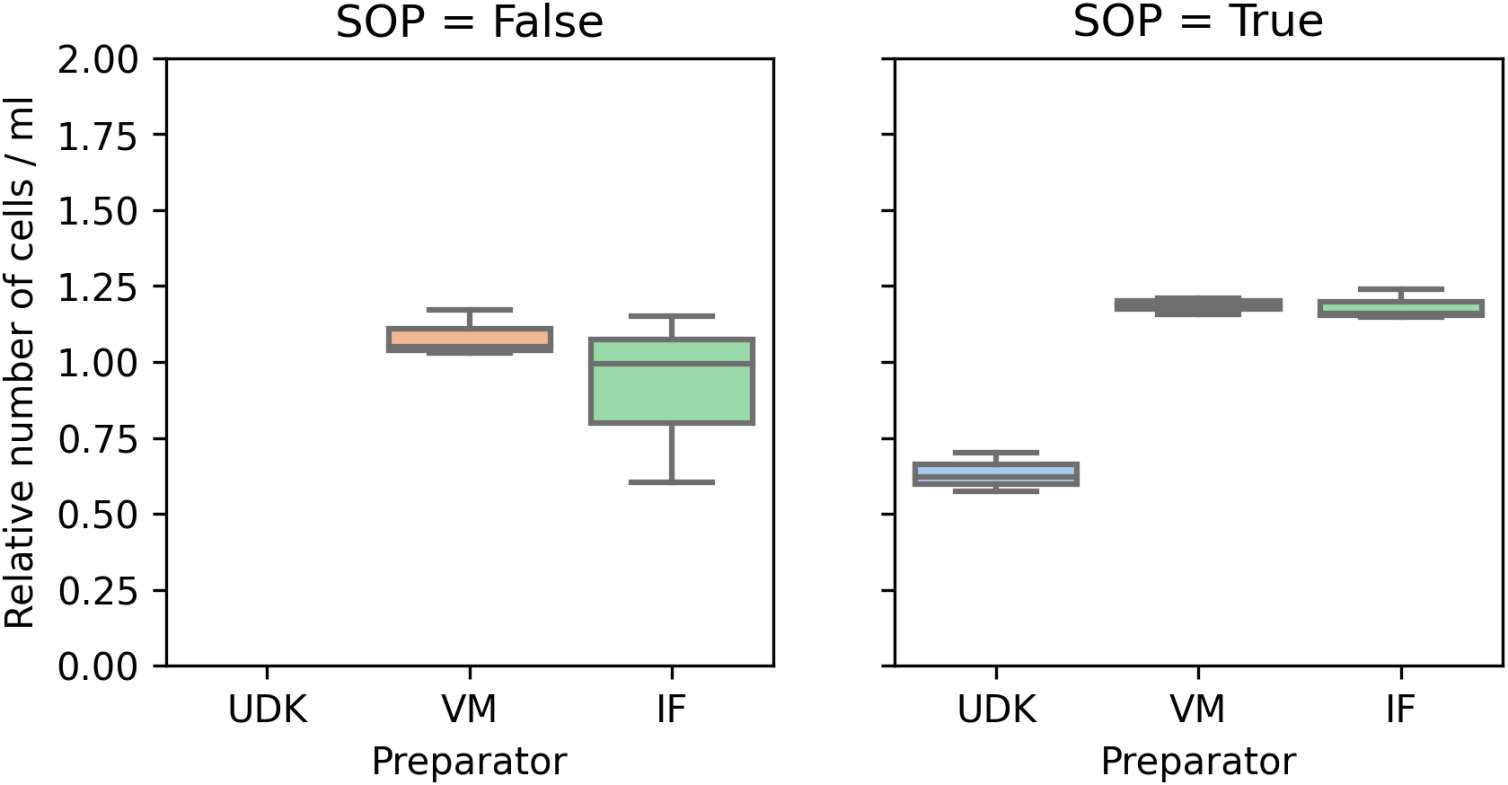
Automatic counting: Here, too, the coefficient of variance in the total number of cells was significantly higher when the experiment was performed without adhering to the SOPs (left). Again, as in Figure 2, the cell counts are scaled to ensure comparability.

Comparing the counts in the two runs, for the two researchers whose cells were viable in the first run, performing the experiment by the SOPs significantly reduced the variance in the counts, both manual and automated. In the first run, the coefficient of variation (cv) for the manual count was in the range 0.16 to 0.24, and 0.07 to 0.30 for the automated counting. In the second run, the cv for manual counting fell to 0.05 to 0.07, and to 0.02 to 0.04 for the automated counting ( Figures 2 and 3).

**Figure 4.**
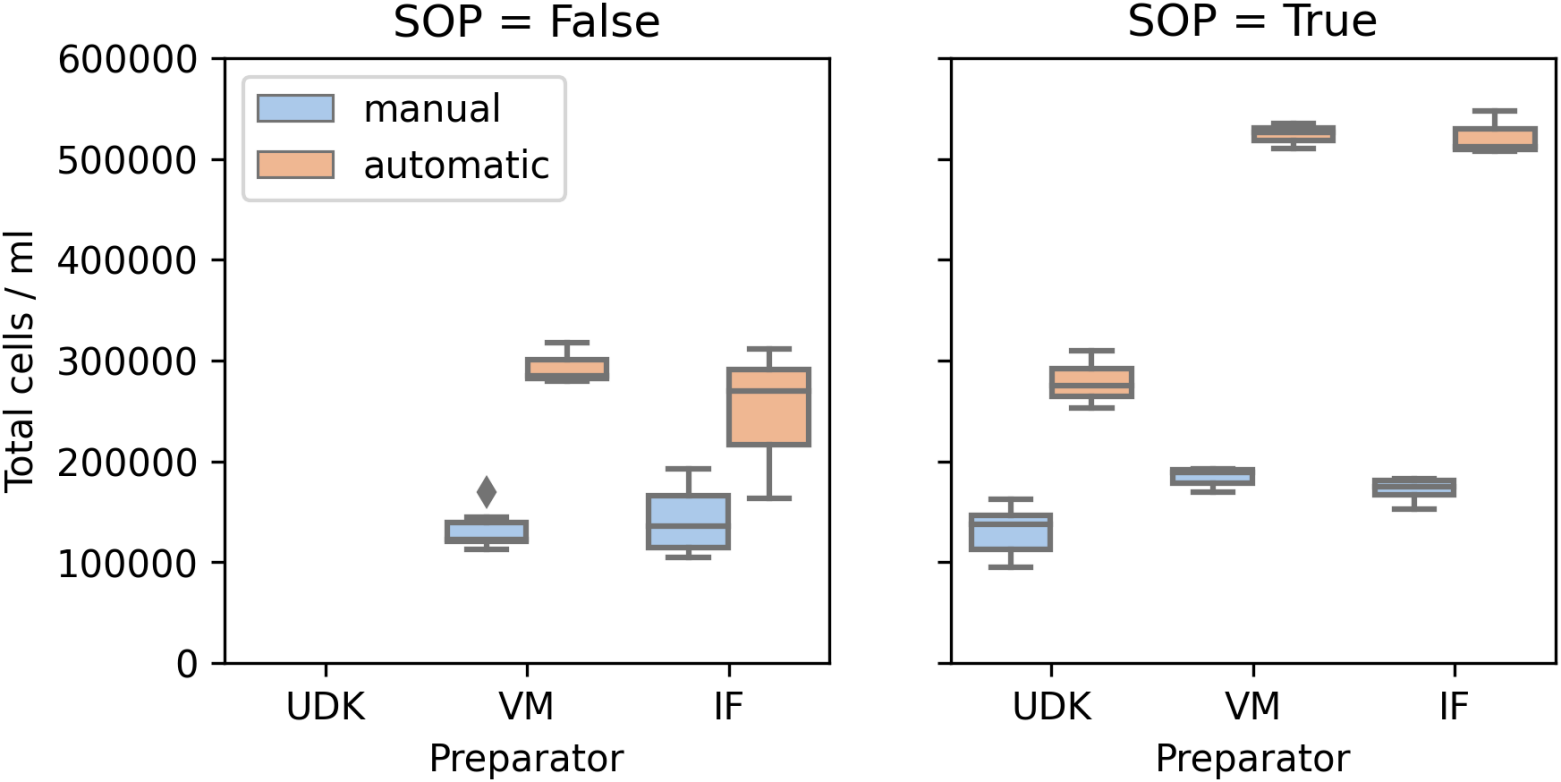
Comparison of the absolute numbers of cells in the solution. Cell Analyzer consistently counts more cells than the researchers using the microscope.

Surprisingly, manual and automated counting led to significantly different numbers, in both runs ( Figure 4). While for the manual counting the total cell numbers were consistently in the range 100,000 – 200,000, the counting machine found 150,000 – 300,000 in the first run and 250,000 – 550,000 in the second. Another point worth noticing is that the number of live and dead cells varied between the machine operators. As the machine requires manual adjustment of threshold for detecting dead cells, we believe this to be one source of variation.

## 4. Discussion

Given the very wide dissemination of the applied experimental procedure, this project presents—to the best of our knowledge—the hitherto first dedicated calculation of sources of variance on cell quantification between automated and manual cell counting in suspension stem cell lines. Although the setup per se is rather simple nature, we are convinced the conclusions are relevant for the general audience.

Research laboratories need to perform cell counting on a daily basis. Precise workflow, accurate execution and reliable measurements within the time constraints are critical parameters for achieving good performance. With this perspective, this study compared two forms of a this widely used basic lab procedure, manual and automated counting.

To evaluate the performance of automated and manual counts, statistical analyses have been made to address the accuracy and repeatability to benchmark one against other method. Careful preparation of the cell culture proved critical. In our case, the experienced researcher (UDK) noticed already during counting that the numbers of live cells were decaying, but this experiment was also specifically set up to identify such problems. The suspicion was confirmed in further counting by the other two researchers (VM, IF). However, due to time and personnel constraints in laboratory practice, multiple counting of a same probe is uncommon. It is therefore conceivable that a less experienced or less attentive (unsuspecting) researcher would fail to notice this problem. Due to exponential cell growth, variations in the starting number of cells grow much larger after incubation. For our manual counting of viable cells, the initial variation of 16% would increase to 44% after three mitoses. The used cell model is in the general range for cell proliferation index of many cancer cells (18–36 hours) and therefore our results may be representative for scenarios when working with other cell models. When the manual counts between different researchers were compared between quadrants, one researcher (IF) counted significantly more dead cells ( *p* < 10 ^−4^). The other two researchers’ guess is that he, due to lack of experience, counted cell debris as dead cells. Adhering to the SOPs led to more consistent results. The second time, the procedure was better prepared and performed more quickly. Although execution speed is not by itself an indication of quality – indeed, it might even be a sign of rushing through the experiment – in case of living cells which might be dying as the experiment is performed, completing it in a timely manner at least ensures that the conditions didn’t change too much between the beginning and the end. Our results seem to confirm this assumption.

Independently of the human operator that prepared the initial cell suspension, we noticed severe difference in absolute quantification of cells (viable and dead) between manual and automatic counting. From our data, this somewhat surprising outcome cannot be explained scientifically. Although our instrument handling and counting setup was performed according manufacturer instructions, it remains possible, albeit somewhat speculative, that the gating setting caused the Cell Analyzer to misclassify debris or cell doublets as single cells. Another likely cause of the differences is the different signal used for identifying the cells: Although both approaches exploit same property of the cells (cell membrane integrity), human counting is based on their colorimetric properties, whereas automatic counting is relies on fluorescence. Since in vitro cell growth assays usually compare different genetic conditions or environmental stressors, absolute quantification is less important than relative quantification between different test conditions. As long as only relative cell growth/cell dying is of concern, experiments can be set up manually and checked for result stability. As long as the same counting method is used when comparing data, both counting methods are equally suitable. This is especially important in lab-to-lab validation, to ensure comparable results.

However, absolute cell number is important in order to define optimal cell plating density at the beginning of the experiment: Sufficient, but not too dense cell-cell contact/confluence grade is a critical factor which impacts the cells’ growth rate, so cells need to be plated in a density that allows exponential growth under control conditions over the entire period of the experiment. Therefore, we suggest to acquire an individual standard growth rate for each cell model to be studied. Our data show that applying the exact same procedure—counting for plating and indirect method for readout at the end of experiment such as MTT/cell TiterGlo—for the “preparatory analysis” and for the actual experiment is very important. In the context of large-sample series, automated counting has well-known benefits, such as reducing the burden on the human operator, not relying on their continuous concentration, and the overall speedup of the experiment. In our small-sample experiment these advantages didn’t apply.

Importantly, adhering the execution to the SOPs, which define maximum time of the execution, familiarization with the SOP, addition of trypsin for a specific time in the incubator and specification of number of times of pipetting for cell separation, led to more stable, in our viewpoint consistent and repeatable results. Although our experiments were performed on a small scale, adhering to the SOPs showed a significant improvement in the stability of the results. We therefore suggest devising such SOPs and enforcing the adherence in wet lab research.
