# Supplementary material for: Reducing sources of variance in experimental procedures in in vitro research
Source: F1000Res. 2022 Jan 7;10:1037. Originally published 2021 Oct 12. [Version 2] doi: 10.12688/f1000research.73497.2 (PMC8749900; doi:10.12688/f1000research.73497.2)
Supplement: Peer review history

# Peer review: Reviewer response for version 2

**DOI:** 10.5256/f1000research.119894.r118914

**Published:** 2022-01-10

**Authors:** Kapil Dave

**Affiliations:** 1 Center for Biophysics and Quantitative Biology, University of Illinois at Urbana-Champaign, Champaign, IL, USA

The authors have addressed most of the concerns highlighted in the earlier review report.

Is the rationale for developing the new method (or application) clearly explained?

Yes

Is the description of the method technically sound?

Partly

Are the conclusions about the method and its performance adequately supported by the findings presented in the article?

Partly

If any results are presented, are all the source data underlying the results available to ensure full reproducibility?

Yes

Are sufficient details provided to allow replication of the method development and its use by others?

Yes

Reviewer Expertise:

Biophysics and Computational Biology, Molecular Dynamics simulations, in vitro experiments, Protein misfolding and aggregation in complex environments

I confirm that I have read this submission and believe that I have an appropriate level of expertise to confirm that it is of an acceptable scientific standard.

# Peer review: Reviewer response for version 1

**DOI:** 10.5256/f1000research.77152.r98468

**Published:** 2021-12-15

**Authors:** Piotr Walczak

**Affiliations:** 1 Department of Diagnostic Radiology and Nuclear Medicine, University of Maryland School of Medicine, Baltimore, MD, USA

This manuscript describes a very simple but widely used technique - counting viable vs. dead cancer cells with the goal of improving reproducibility of the method. I suggest correcting the following:

- For cell preparation, please specify what the total volume was from which 1ml was drawn three times.
- Was there mixing involved before/after or in between the individual sampling to avoid sedimentation?
- It is not clear what the authors mean by "cells were dying rapidly during counting". If that was the case, it indicates some unspecified error which was corrected when they used their specific SOP. They should at least attempt explaining what may have caused this unexpected result. 
- Differences between the "casual" and SOP-based counting should be described at least briefly in the main manuscript, not only in the supplementary data. 
- Photographic images of cells during counting with markings what was considered as live vs. dead cells for both manual and automatic methods would be helpful. 

Is the rationale for developing the new method (or application) clearly explained?

Yes

Is the description of the method technically sound?

Yes

Are the conclusions about the method and its performance adequately supported by the findings presented in the article?

Partly

If any results are presented, are all the source data underlying the results available to ensure full reproducibility?

Yes

Are sufficient details provided to allow replication of the method development and its use by others?

Yes

Reviewer Expertise:

Image-guided drug delivery to the brain

I confirm that I have read this submission and believe that I have an appropriate level of expertise to confirm that it is of an acceptable scientific standard.

# Peer review: Reviewer response for version 1

**DOI:** 10.5256/f1000research.77152.r98466

**Published:** 2021-11-08

**Authors:** Kapil Dave

**Affiliations:** 1 Center for Biophysics and Quantitative Biology, University of Illinois at Urbana-Champaign, Champaign, IL, USA

In this manuscript, the authors have demonstrated that following and promoting a standard procedure for conducting a wet lab experiment results in lower variation. This approach of documenting and following a standard protocol can help alleviate the lab-to-lab repeatability problems. Overall, the data supports the conclusion. However, the authors should rewrite the discussion section (details below) for the readers to follow the results, along with addressing the comments below: 

- Page 3, "distinguishing in size or morphology of blood cell types": Please remove the "in" from this sentence. Please carefully proofread the article to correct similar errors in the paper and avoid using ambiguous English language.
- Page 3, "A senior, experienced researcher (UKD)": the term UKD is used in the text, while in the figure, legend, and caption "UDK" is used. Please use consistent terminology across the manuscript.
- Figure 1, SOP=False: The author should clarify why the UDK operator only showed high variation in viable cell count, whereas an operator with no prior wet-lab experience (IF) had lower variability with the same suspension.
- Page 4, Section 3 "Consequently…… For further analysis, we excluded this first suspension and used a different cell culture to prepare new solutions": it is not clear from this paragraph which "new solutions" that were prepared were made by which human operator.
- Page 5, "A similar pattern, with an even…. cv = 0.168": the paragraph is vague and not clearly written, authors, should re-write to make it clear to the readers. I suggest making an apples-to-apples comparison between counting viable cells (manual vs automated) and Dead cells (manual vs automated).
- Page 6, Section 4: Please rewrite the paragraph " Research laboratories ….. Cell quantification" - it's not clear what two forms the authors are referring to in this paragraph.
- Page 7, "The second time, the procedure was better prepared and performed more quickly, this indicates that the duration of the experiment plays a role”: Authors should clarify or provide more evidence to support this statement. Performing an experiment faster doesn’t always guarantee less variation (shows familiarity), rather, following the SOP diligently could help reduce variation.

Is the rationale for developing the new method (or application) clearly explained?

Yes

Is the description of the method technically sound?

Partly

Are the conclusions about the method and its performance adequately supported by the findings presented in the article?

Partly

If any results are presented, are all the source data underlying the results available to ensure full reproducibility?

Yes

Are sufficient details provided to allow replication of the method development and its use by others?

Yes

Reviewer Expertise:

Biophysics and Computational Biology, Molecular Dynamics simulations, in vitro experiments, Protein misfolding and aggregation in complex environments

I confirm that I have read this submission and believe that I have an appropriate level of expertise to confirm that it is of an acceptable scientific standard, however I have significant reservations, as outlined above.

# Response

**Published:** 2021-12-29

**Authors:** Igor Fischer

Thank you very much for careful reading and providing constructive suggestions to imporve the article.

 1. We've corrected the error, carefully proofread and extensively edited the article.

 2. We corrected the typo and ensured consistent usage of abbreviations.

 3. In the caption to the Figure 1, we clarified that the high volatility was observed in the first solution that was prepared ("the first prepared" inserted). In the text ("Results", p.4), we added the sentences "Although the way the experiment was conceived did not allow for establishing the cause with certainty, we assume that a toxic agent - perhaps the alcohol used for cleaning the equipment - somehow contaminated the culture" and "Special care was taken to avoid contamination".

 4. We clarified the operators and the order in which they prepared the solutions: "first suspension ... (prepared by UDK)" and "The second solution was prepared by VM and the third by IF"

 5. We rewrote the paragraph as suggested and included numbers for dead cells.

 6. We rewrote the paragraph as suggested, to read: "Research laboratories need to perform cell counting on a daily basis. Precise workflow, accurate execution and reliable measurements within the time constraints are critical parameters for achieving good performance. With this perspective, this study compared two forms of a this widely used basic lab procedure, manual and automated counting."

 7. We added a clarification, so that the paragraph now ends with: "Although execution speed is not by itself an indication of quality – indeed, it might even be a sign of rushing through the experiment – in case of living cells which might be dying as the experiment is performed, completing it in a timely manner at least ensures that the conditions didn’t change too much between the beginning and the end. Our results seem to confirm this assumption."
